# Prophylactic Antibiotic Use in Transperineal Ultrasound-Guided Prostate Biopsy Under Local Anesthesia: A Retrospective Observational Study

**DOI:** 10.7759/cureus.94459

**Published:** 2025-10-13

**Authors:** Anurag Agarwal, Katherine V Rhodes, Daniel Grogan, Nurul Aimi B Ismail, Ashok Kailasa, Vignesh Balasubaramaniam, Krassen E Donev

**Affiliations:** 1 General Surgery, Betsi Cadwaladr University Health Board, Bangor, GBR; 2 Urology, Betsi Cadwaladr University Health Board, Bangor, GBR

**Keywords:** prophylactic antibiotics, prostate biopsy, prostate cancer, transperineal biopsy, uti

## Abstract

Aim: Transperineal prostate biopsy under local anesthesia (LATP) carries a low risk of infection, yet prophylactic antibiotics remain commonly used. This study evaluated infection outcomes with and without antibiotic use during LATP.

Methods: We conducted a retrospective analysis of 186 men undergoing LATP between February 2022 and June 2025 under the care of a single urologist. Patients either received a single prophylactic antibiotic dose (Group 1, n = 91) or no antibiotics (Group 2, n = 95). The primary outcome was urinary tract infection (UTI) within 30 days post-biopsy; secondary outcomes included hospital admissions for infection.

Results: Baseline characteristics were similar between groups. The incidence of post-biopsy UTI was low and did not differ significantly between patients who received antibiotics and those who did not. No cases of sepsis occurred. Hospital admissions for infection-related complications were rare in both groups. A significant association was observed between smoking history and risk of UTI (p < 0.05), whereas other comorbidities showed no association.

Conclusions: In this cohort, the omission of prophylactic antibiotics during LATP prostate biopsy was not associated with an increased risk of infection. These findings support the safety of a no-antibiotic approach and highlight smoking as a potential risk factor for post-biopsy UTI. Broader adoption of selective antibiotic protocols may contribute to improved antimicrobial stewardship without compromising patient safety.

## Introduction

Prostate cancer is the most common cancer diagnosis in men, with more than 1.4 million new cases annually worldwide, and remains a significant leading cause of cancer-related mortalities [1,2]. Accurate diagnosis and risk stratification are based on histopathologic examination obtained by prostate biopsy, traditionally accomplished through a transrectal approach by using ultrasound guidance. Although this technique is technically straightforward and accessible to everyone, it necessitates passage through the rectal mucosa, resulting in contamination by rectal flora [3,4]. Consequently, infectious complications are among the most significant risks of transrectal biopsy, with sepsis rates of 1%-3% and increasing rates of multidrug-resistant organisms contributing to this issue [5].

To mitigate infectious risks, antibiotic prophylaxis has become a standard component of transrectal biopsy regimens. Fluoroquinolones have been the drug of choice for decades due to their coverage and prostatic tissue penetration [5]. However, the global rise in fluoroquinolone resistance, along with safety concerns regarding their adverse effect profile, has prompted reconsideration of this practice [6]. The guidelines now recommend alternative therapy or directed prophylaxis based on rectal swab culture, yet infections, including cases requiring hospitalization for sepsis, remain a troubling complication [7,8].

To alleviate these problems, the transperineal approach to prostate biopsy has experienced renewed popularity. By gaining access to the prostate via the perineum, the procedure avoids altogether piercing the rectal mucosa, therefore greatly reducing the risk of introducing gastrointestinal flora into the urinary system or bloodstream. Initially performed under general or spinal anesthesia, technical advancements and utilization of local anesthesia methods have made transperineal biopsy increasingly feasible in outpatients. Several studies have demonstrated remarkably low rates of infectious complications with the transperineal approach, even without the use of prophylactic antibiotics [5,9].

Despite a favorable safety record, the majority of institutions continue to administer prophylactic antibiotics as a precaution with local anesthesia transperineal (LATP) prostate biopsy. The practice is largely extrapolated from recommendations formulated for transrectal biopsy and continues to be practiced in the absence of compelling evidence to comment particularly on the use of antibiotics in the transperineal environment. The impact of continuous prophylaxis extends beyond patient-level safety; overuse of antibiotics feeds the broader problem of antimicrobial resistance and exposes patients to risks of adverse drug reactions [10].

Current guidelines offer varying recommendations, reflecting the heterogeneity of the evidence base. The European Association of Urology acknowledges the low risk of infection with transperineal biopsy but stops short of opposing universal prophylaxis, suggesting instead that local practice be guided by institutional experience and local resistance patterns [10,11]. Similarly, other professional societies recognize the potential to omit antibiotics in selected patients but call for more definitive studies to establish best practices [12].

In this study, we sought to evaluate the role of prophylactic antibiotics in patients undergoing LATP prostate biopsy. We examined infection-related outcomes in our institution’s cohort of men who underwent this procedure, comparing those who received prophylaxis with those managed without antibiotics. We aimed to assess whether withholding routine antibiotic prophylaxis in the context of LATP is associated with an increased risk of infectious complications. By addressing this question, we hope to inform clinical decision-making and contribute to evolving efforts to balance patient safety with responsible antibiotic use.

## Materials and methods

Study design and setting

This was a retrospective observational study conducted in the Department of Urology, Betsi Cadwaladr University Health Board (BCUHB). The study evaluated infectious outcomes in patients undergoing LATP prostate biopsy during two distinct periods with differing institutional protocols for antimicrobial prophylaxis. All procedures were performed by a single consultant urologist, ensuring consistency in biopsy technique and clinical decision-making.

The change in antibiotic prophylaxis was not introduced for the purposes of this audit but resulted from a change in practice aimed at aligning with emerging evidence and antimicrobial stewardship principles. Until March 2023, standard practice included a single intravenous dose of gentamicin prior to biopsy as per local hospital guidelines [13]. From March 2023 onward, prophylaxis was no longer routinely administered for LATP biopsies. No other procedural changes occurred during the study period.

Study population

We included all consecutive adult male patients who underwent LATP prostate biopsy between February 1, 2022, and June 30, 2025. All procedures were performed by a single consultant urologist to ensure consistency in technique. Patients were classified into two groups according to the institutional protocol in effect at the time of their procedure: Group 1 (prophylaxis group; February 2022 to March 2023) received a single pre-procedure intravenous dose of gentamicin, and Group 2 (no-prophylaxis group; March 2023 to June 2025) underwent biopsy without antibiotic prophylaxis. There was no overlap between the study periods.

Data collection

Data were collected through a structured, multi-step process to ensure accuracy and completeness. First, all LATP biopsy cases were identified using theater procedural logs. These cases were then linked with hospital electronic health records (EHRs) and laboratory databases to enable comprehensive data capture. Trained reviewers systematically extracted demographic information, comorbidities, medication use, and procedural details. Demographic variables included patient age, smoking status, and family history of prostate cancer. Medical history encompassed hypertension (HTN), type 2 diabetes mellitus (T2DM), coronary heart disease (CHD), and cerebrovascular accident (CVA). Medication exposure was assessed with particular attention to finasteride use prior to biopsy. Pre-procedural testing included urine dipstick analysis and midstream urine (MSU) culture results. Post-procedural outcomes recorded were urinary tract infection (UTI) or sepsis within 30 days, hospital admissions, and follow-up urine results when available. All suspected infectious complications were rigorously validated through a detailed review of microbiology reports, clinical documentation, and discharge summaries to ensure consistency and reliability of outcome ascertainment.

Outcome measures

The primary outcome was the incidence of UTI within 30 days of biopsy, defined as the presence of urinary symptoms (dysuria, urgency, frequency, or fever) accompanied by a positive urine culture requiring antibiotic treatment. Secondary outcomes included the incidence of sepsis within 30 days, all-cause hospital admission within 30 days, and detection of asymptomatic bacteriuria following the procedure.

Statistical analysis

Baseline characteristics were summarized as means with standard deviations or medians with interquartile ranges for continuous variables and as frequencies with percentages for categorical variables. Comparisons between groups were made using the chi-square test or Fisher’s exact test for categorical variables. Statistical significance was defined as a two-tailed p < 0.05. Analyses were performed using IBM SPSS Statistics for Windows, Version 31.0 (Released 2025; IBM Corp., Armonk, NY, USA).

Ethics statement

The study was approved by the Institution Clinical Audit Team (Project ID: 2290). Given its retrospective design, informed consent was waived. All data were anonymized prior to analysis. As this was an audit of existing practice, no changes were made to clinical care pathways.

## Results

A total of 186 patients were included in the analysis: 91 patients in Group 1 (who received prophylactic antibiotics) and 95 patients in Group 2 (who did not receive antibiotics). Baseline demographic and clinical characteristics were similar between groups (Table 1). The mean age was 69.99 years (SD, 7.456) in Group 1 and 68.28 years (SD, 9.74) in Group 2. The prevalence of HTN, T2DM, CHD, CVA, family history of prostate cancer, and pre-procedure finasteride use did not differ significantly between groups except for smoking history, where 64.8% (n= 59) from Group 1 and 80% (n= 76) from Group 2 had never smoked, as shown in Table 1.

**Table 1:** Demographic variables This table summarizes the baseline demographic and clinical characteristics of patients who either received prophylactic antibiotics (Group 1) or did not receive antibiotics (Group 2) prior to LATP biopsy. Continuous variables (age and PSA) are expressed as mean (standard deviation), while categorical variables are presented as the number of patients with percentages in parentheses. The chi-square test was used for statistical analysis, and p < 0.05 was considered statistically significant. *Values are depicted as mean (standard deviation).

Particulars	Group 1 (received antibiotics), n (%)	Group 2 (did not receive antibiotics), n (%)	p-value
Age*	69.99 (7.4)	68.28 (9.7)	
Family history of prostate cancer	16 (17.6)	21 (22.1)	0.44
Prior history of finasteride intake	6 (6.6)	6 (6.3)	0.93
Prostate-specific antigen (PSA) levels*	77.77 (562.22)	24.46 (60.27)	
Smoking			
Current	7 (7.7)	7 (7.4)	0.03
Ex-smoker	25 (27.5)	12 (12.6)	
Non-smoker	59 (64.8)	76 (80.0)	
Comorbidities			
Hypertension	34 (37.4)	40 (42.1)	0.50
Type 2 diabetes mellitus	9 (9.9)	11 (11.6)	0.71
Cardiovascular accidents	6 (6.6)	3 (3.2)	0.27
Coronary heart disease	11 (12.1)	14 (14.7)	0.59

The overall incidence of UTI within 30 days of biopsy was low in both groups. In Group 1 (with antibiotics), one patient (n = 1, 1.9%) developed UTI requiring oral antibiotic treatment. In Group 2 (no antibiotics), four patient (n = 4, 4.2%) developed UTI (p = 0.190). Only one case (n = 1, 1%) of sepsis or hospital admission for infection was observed in Group 2, whereas no case of sepsis was observed in Group 1, as depicted in Table 2.

**Table 2:** Incidence of post-procedural infections and hospital admissions This table presents the incidence of post-procedural infections and hospital admissions following LATP biopsy, stratified by whether patients received prophylactic antibiotics (Group 1) or not (Group 2). Post-procedural midstream urine (MSU) culture results were categorized as infection present or absent, while hospital admissions were further classified by cause (hematuria, urinary retention, or sepsis). Values are reported as the number of patients with percentages in parentheses. The chi-square test was used for statistical analysis and a p-value of <0.05 was considered statistically significant.

Particulars	Group 1 (received antibiotics), n (%)	Group 2 (did not receive antibiotics), n (%)	p-value
Post-MSU infection			
Yes	1 (1.9)	4 (4.2)	0.19
No	90 (98.1)	91 (95.8)	
Admission to the hospital			
Hematuria	0 (0)	2 (2.1)	0.29
Urinary retention	2 (2.2)	1 (1)	
Sepsis	0 (0)	1 (1)	

Smoking history was significantly associated with an increased risk of developing post-biopsy UTI (p = 0.036). No other patient factors, including T2DM, BMI, CVA, CHD, family history of prostate cancer, previous biopsy, or finasteride use, were significantly associated with infection risk.

## Discussion

In this retrospective study comparing patients undergoing LATP prostate biopsy with or without prophylactic antibiotics, no significant difference in post-biopsy UTI rates between the two groups was found. The overall incidence of infection was low and comparable in both groups, consistent with prior reports demonstrating the low infectious risk associated with the transperineal approach. Importantly, one patient (1%) in Group 2 required hospital admission due to UTI. Two patients in Group 1 were admitted because of a procedure-related complication, urinary retention (n = 2, 2.2%), while four patients (4.2%) in Group 2 required admission following the procedure, as shown in Table 2.

These findings align with emerging evidence suggesting that prophylactic antibiotics may not be necessary in LATP biopsy [14,15]. Grummet et al. reported low rates of infection in patients undergoing transperineal biopsy without antibiotic treatment, highlighting the procedure’s inherently low contamination risk due to its avoidance of the rectal mucosa [6]. Similarly, Basourakos et al. and Jacewicz et al. have not observed a statistically significant difference in UTI rates between patients receiving and not receiving prophylaxis in the transperineal setting [5,16]. A growing number of institutions have begun to adopt no-antibiotic protocols for LATP biopsy in alignment with antimicrobial stewardship programs, without a corresponding rise in infectious complications [17,18].

While most baseline factors were not associated with infection risk in our study, we observed a significant association between smoking history and post-biopsy UTI. Patients who developed UTI were more likely to be current or former smokers than those who did not smoke. This finding may reflect the impact of smoking on mucosal immunity, urothelial integrity, or systemic inflammatory responses, though the exact mechanism remains unclear. To our knowledge, this association has not been widely reported in previous LATP biopsy studies, and it warrants further investigation in larger cohorts [19].

Our results support the safety of omitting prophylactic antibiotics in LATP prostate biopsy and add to the growing evidence that such protocols can safely be implemented in everyday practice. With the global push to reduce unnecessary antibiotic use, particularly in procedures with a low infection risk profile, the adoption of no-antibiotic LATP biopsy protocols may be a valuable opportunity to contribute to antimicrobial stewardship. Further prospective studies on larger sample sizes are required to validate these findings and to investigate the potential role of patient-level risk factors, such as smoking, in the prediction of infectious complications.

Strengths and limitations

The strengths of our study include clearly defined cohorts, a standardized biopsy technique, and comprehensive post-procedure follow-up. However, several limitations merit consideration. The retrospective design limits causal inference, and the modest number of UTI events restricts the power of subgroup analyses. Additionally, post-procedure urine testing was not universally performed, and some cases of asymptomatic bacteriuria may have gone undetected. Moreover, patients who developed UTI or progressed to sepsis but did not present to their GP or a hospital may have been missed. Nevertheless, our region’s integrated EHR system captures data from all three local hospitals, which reduces the risk of missing clinically significant events. Importantly, such infections were rare and equally distributed between groups.

## Conclusions

In this retrospective observational study, the omission of prophylactic antibiotics during LATP prostate biopsy was not associated with an increased risk of urinary tract infection. The overall infection rate was low and comparable between patients who received antibiotics and those who did not. Smoking history was significantly associated with post-biopsy UTI, suggesting a potential modifiable risk factor that warrants further investigation. These findings support the safety of a no-antibiotic approach in LATP biopsy and reinforce the importance of individualized risk assessment and antimicrobial stewardship in urological practice.
